# Regional disparities in maternal and child health indicators: Cluster analysis of districts in Bangladesh

**DOI:** 10.1371/journal.pone.0210697

**Published:** 2019-02-06

**Authors:** Enayetur Raheem, Jahidur Rahman Khan, Mohammad Sorowar Hossain

**Affiliations:** 1 Biomedical Research Foundation, Dhaka, Bangladesh; 2 Centre for Research and Action in Public Health, Health Research Institute, Faculty of Health, University of Canberra, Bruce, Australian Capital Territory, Australia; TNO, NETHERLANDS

## Abstract

Efforts to mitigate public health concerns are showing encouraging results over the time but disparities across the geographic regions still exist within countries. Inadequate researches on the regional disparities of health indicators based on representative and comparable data create challenges to develop evidence-based health policies, planning and future studies in developing countries like Bangladesh. This study examined the disparities among districts on various maternal and child health indicators in Bangladesh. Cluster analysis–an unsupervised learning technique was used based on nationally representative dataset originated from Multiple Indicator Cluster Survey (MICS), 2012–13. According to our results, Bangladesh is classified into two clusters based on different health indicators with substantial variations in districts per clusters for different sets of indicators suggesting regional variation across the indicators. There is a need to differentially focus on community-level interventions aimed at increasing maternal and child health care utilization and improving the socioeconomic position of mothers, especially in disadvantaged regions. The cluster analysis approach is unique in terms of the use of health care metrics in a multivariate setup to study regional similarity and dissimilarity in the context of Bangladesh.

## Introduction

Representative and comparable health information within a country is fundamental for the development of evidence-based health policies, planning educational and awareness programs, and designing public health studies. Most public health related studies in developing countries are predominantly based on limited communities selected for convenience because of favorable historical, financial, or geographic circumstances. Unfortunately, these data from selected populations or communities are extrapolated or generalized to entire population through judgment [1]. In this perspective, multivariate cluster analysis (also known as unsupervised learning technique) could be a holistic approach to study the regional disparities (similarity or dissimilarity) within a developing country or among developing countries based on available health indicators or metrics. Clusters of regions can be created based on single or multiple factors. We utilized multiple indicators to form the clusters, and hence we refer to it as multivariate cluster analysis. Cluster analysis is widely used in social sciences [2] and commercial market analysis research [3], and it is being applied in epidemiological and public health studies as well [4]. The unsupervised learning technique is generally used to discover hidden structures in the data where the right groups are not known upfront. The goal is to find natural clusters or patterns in the data so that elements in the same cluster are similar to each other than items in a different cluster [5].

Bangladesh is a developing country with a population over 160 million. For administration purpose, Bangladesh is divided into 64 districts under eight divisions. Each district is further subdivided into smaller administrative units such as upazilla (sub-district), municipalities and union councils. The motivation of this study was to develop a framework where the regions of a country can be grouped based on some key indicators such as maternal and child health. If we find that some regions share a similar profile based on the chosen indicators, this information could be used for resource planning and allocation. We could also utilize the clustering pattern among the regions to better design cost effective surveys for evaluating interventions. In other words, a community level finding could potentially be generalized to a much broader geographic spectrum that share similar profiles.

There were two reasons for doing a cluster analysis. First, the government reports provided univariate results. Second, this method would confirm results that are otherwise perceived to be true. In China, for instance, this type of analysis to study the regional distribution of foreign direct investment (FDI) revealed that Guangdong province had the highest level of FDI which was consistent with its perceived economic development [6].

The aim of this study was to use cluster analysis techniques to determine if there are homogeneous groups among the districts based on maternal, child health, and some socio-demographic characteristics.

## Materials and methods

### Data and variables

Bangladesh Multiple Indicator Cluster Survey (MICS) 2012–2013 data was used in the present study. The survey was conducted in 51895 households by the Bangladesh Bureau of Statistics (BBS) over a period from December 2012 to April 2013 [7]. MICS 2012–2013 survey report represents maternal and health conditions of children in Bangladesh based on 79 indicators grouped under 10 categories related to child mortality, nutrition and breastfeeding, child health, access to safe drinking water and improved sanitation, reproductive health, child development, literacy and education, child protection, HIV/AIDS awareness, and access to mass media and ICT [8]. Majority of the indicators were measured in terms of percentages. A subset of indicators was first selected based on their uniqueness. Then, standard deviation of each of the indicators was calculated. Indicators with a standard deviation greater than five were retained to capture variations in the data. Demographic variables were extracted from 2011 Bangladesh Population Census Report [9].

### Method of analysis

In the following, we briefly illustrate cluster analysis technique. District level statistics on the following indicators were extracted from MICS report: underweight prevalence (moderate and severe), stunting prevalence (moderate and severe), wasting prevalence (moderate and severe), overweight prevalence, and iodized salt consumption. Then cluster analysis was performed using different methods of clustering such as “hierarchical”, “kmeans”, “pam”, among others. Different algorithms were tried with number of clusters ranging from 2 to 7. We used silhoutte plot to determine the number of clusters [10] using the R package cluster [11]. The cluster selection was validated based on internal validation and stability measures using cValid R package [12–14].

## Results

**Fig 1:**
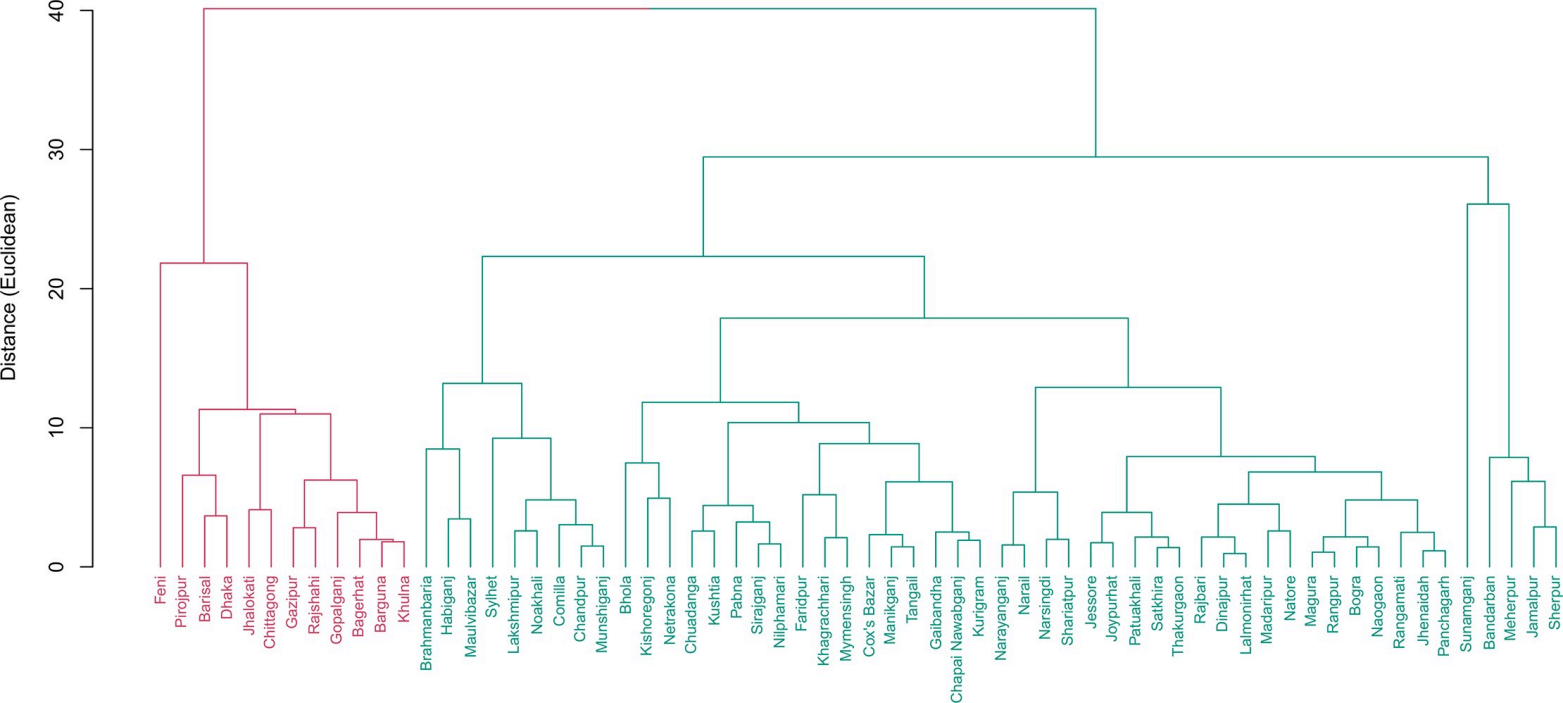
Cluster of districts based on demographic indicators. Two clusters were found based on demographic indicators. Cluster 1 includes 12 districts while 52 districts were in cluster 2. Districts in cluster 1 have 10% point higher literacy rate than the national rate of 63%.

Table 1 represents cluster averages of districts and division-level averages of two separate sets of indicators–“demographic indicators” and “literacy and educational indicators”. Based on five demographic indicators, two clusters were formed where cluster 1 comprised of 12 districts and cluster 2 had 52 remaining districts (Fig 1). Two clusters were different in the literacy rate (7+years). The larger cluster had literacy rate (60.3%), which was close to the national rate (63%), while the smaller cluster had literacy rate that is more than 10% point higher compared to the national rate.

**Table 1:** Cluster averages of districts along with averages of the divisions and Bangladesh as a whole based on demographic indicators and literacy and educational indicators.

	Cluster Average									
**Indicators**	**Cluster 1**	**Cluster 2**								
	**12 districts**	**52 districts**	**BAR**	**CTG**	**DHK**	**KHL**	**RAJ**	**RNG**	**SYL**	**BD**
Percent male-headed households	87.7	88.7	90.8	82.9	87.8	91	90.8	91.1	86.7	88.5
Average household size	4.4	4.4	4.5	4.8	4.3	4.1	4.0	4.1	5.1	4.4
Literacy rate (7+ years)	75.1	60.3	71.8	63.6	61.5	63.8	61.9	60.2	60.7	63
Unemployment rate	9.6	9.7	9.9	12.2	9.8	6.7	6.9	7.2	21.5	9.8
Population density per square km	1549	957	655	990	1720	803	1007	958	775	1108
	**18 districts**	**46 districts**	**BAR**	**CTG**	**DHK**	**KHL**	**RAJ**	**RNG**	**SYL**	**BD**
Literacy among young women (15–24 years)	80.42	84.0	86.1	81.5	80.9	87.8	83.0	80.0	78.2	82.0
Primary school net attendance ratio (adjusted)	71.48	76.95	71.9	72.4	72.5	75.4	75.1	75.7	69.4	73.2
Secondary school net attendance ratio (adjusted)	43.47	53.27	47.8	45.6	45.6	50.9	46.1	52.3	32.7	46.1
Primary completion rate	73.6	103.33	79.5	80.8	71.2	82.4	92.5	87.4	73.5	79.5

BAR, Barisal; CTG, Chittagong; DHK, Dhaka; KHL, Khulna; RAJ, Rajshahi; RNG, Rangpur; SYL, Sylhet; BD, Bangladesh.

**Fig 2:**
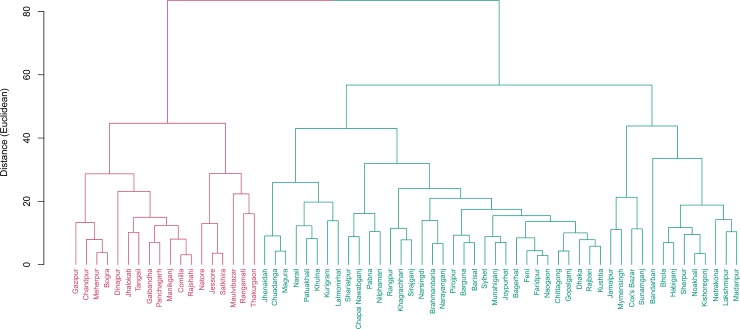
Cluster of districts based on educational indicators. Two clusters were found. Cluster 1 include 18 districts while 46 districts were in cluster 2. Districts in cluster 1 are performing below national level whereas those in cluster 2 are above national level.

Similarly, two clusters were formed for the literacy and education related indicators with 18 districts in one cluster and 46 districts in the second cluster (Fig 2). On an average the literacy rate among young women (15–24) were higher in the second cluster with 46 districts than the national average. Notably, completion of primary education rate in these districts was above 100 indicating much older student population were completing primary grade in these districts compared to the students of primary school age in the same cluster (Table 1).

**Fig 3:**
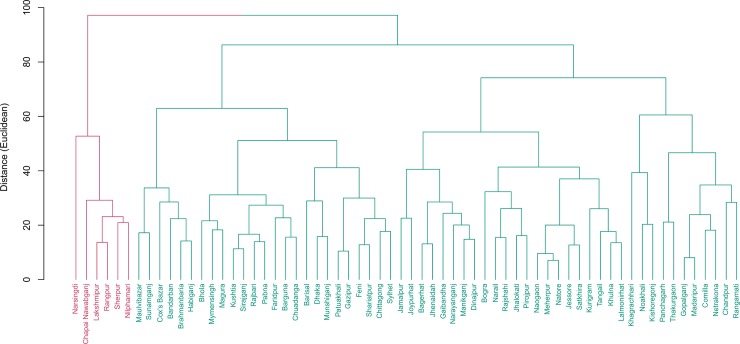
Cluster of districts based on child protection and development indicators. Two clusters were found. Cluster 1 include 58 districts while 6 districts were in cluster 2. Percentage of women married before age 15 is 34.2 in cluster 2 which is 10 percentage point higher than national average (23.8%).

Table 2 and Fig 3 represent clusters and cluster averages for child development and child protection indicators. In cluster 2, percentage of children (36 to 59 months age-group) attending early childhood education was much higher than national average (32% vs 13.4%). Not surprisingly, these six districts had fewer children of the same age group getting adult support (51.5% vs 78% nationally). The contrasting picture made sense since lack of adult support for learning was being supplemented by early childhood education. This result served as a validation of the clustering algorithms used in this study.

**Table 2:** Cluster averages of districts along with averages of the divisions and Bangladesh as a whole based on child protection and development indicators.

Indicators	Cluster Average									
	Cluster 1	Cluster 2								
	58 districts	6 districts	BAR	CTG	DHK	KHL	RAJ	RNG	SYL	BD
Attended early childhood education (36–59 months)	11.8	32	18.4	11.7	15.6	13.6	10.2	13.2	10.5	13.4
Getting adult support for learning (36–59 months)	79.5	51.5	80.4	76.5	78.9	85.9	75.1	75	76.4	78
Early development index	64.8	64.8	67.3	54.4	65.1	69	65.2	77.7	54	63.9
Percentages of birth registration (under 5)	38.5	26.2	32.3	41.4	34.3	32.1	32.6	47.6	35	37
Women (15–49) married before 15	23.2	34.2	20.8	14.5	23.1	31.1	33.3	31.5	9.2	23.8
Girls 15–19 currently married	35.4	36.0	31.7	27.7	33.3	43.5	47.8	41.9	13.7	34.3

BAR, Barisal; CTG, Chittagong; DHK, Dhaka; KHL, Khulna; RAJ, Rajshahi; RNG, Rangpur; SYL, Sylhet; BD, Bangladesh.

**Fig 4:**
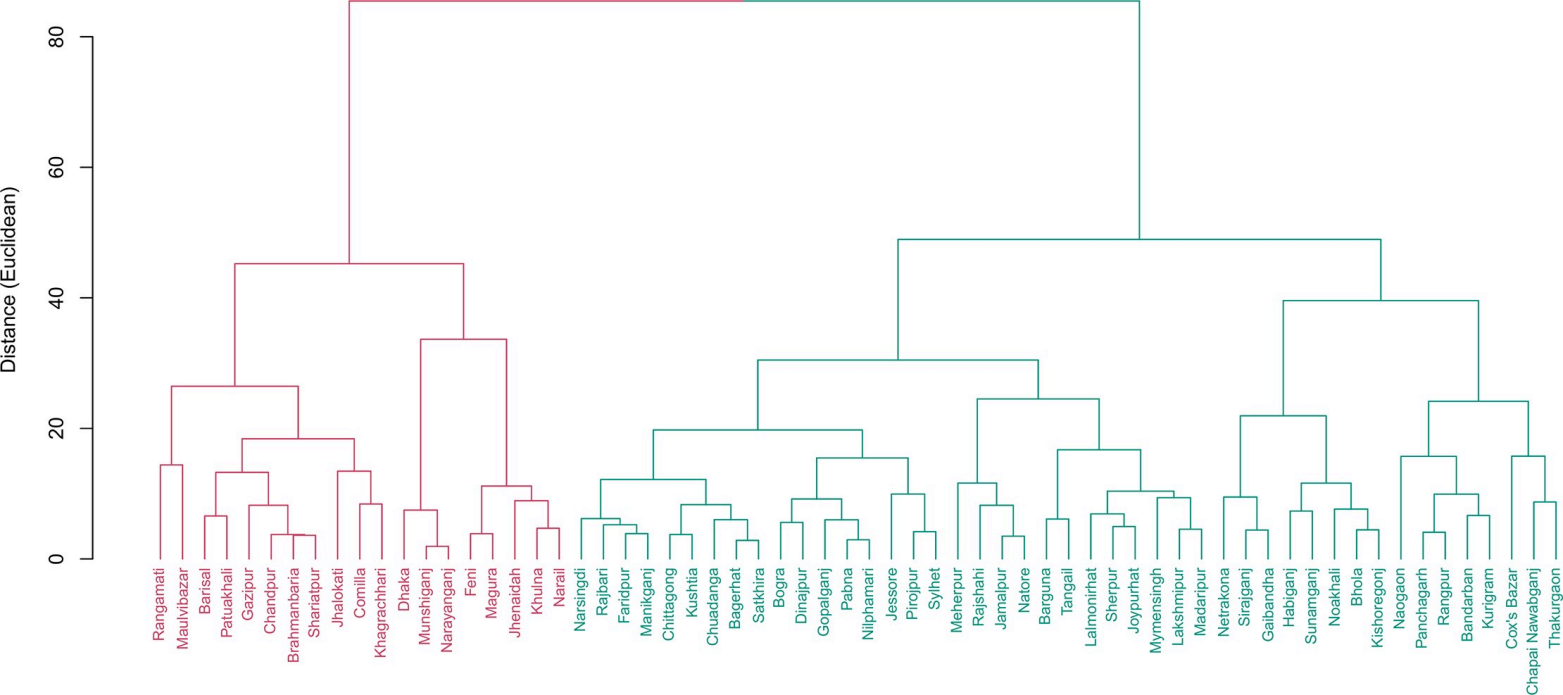
Cluster of districts based on nutrition indicators. Two clusters were found. Cluster 1 include 45 districts while 19 districts were in cluster 2.

Table 3 and Fig 4 represent clusters and cluster averages for the nutritional indicators. Overweight prevalence (1.8% on average) was higher in the better performing districts (cluster 2) compared to the underperforming districts (1.4% on average) as well as national level (1.6%). For the breastfeeding category, the percentage of babies in cluster 2 (11 districts) breastfed within one hour of birth was nearly half at 33.9% compared to the national level of 57.4% (S1 Table, S1 Fig).

**Table 3:** Cluster averages of districts along with averages of the divisions and Bangladesh as a whole based on nutrition indicators.

Indicators	Cluster Average									
	Cluster 1	Cluster 2								
	45 districts	19 districts	BAR	CTG	DHK	KHL	RAJ	RNG	SYL	BD
Underweight prevalence (moderate and severe)	32.3	30.3	35.2	32.2	30.8	28.5	29.9	32.6	39.7	31.9
Stunting prevalence (moderate and severe)	41.5	40.5	41.4	43.1	42.1	34.4	39.4	43.7	50.6	42.0
Wasting prevalence (moderate and severe)	10.0	9.4	11.7	9.2	9.2	10.0	9.1	8.7	13.3	9.6
Overweight prevalence	1.4	1.8	0.8	1.2	2.2	1.1	1.2	1.1	2.9	1.6
Iodized salt consumption	42.3	74.7	62.4	59.3	64.6	60.1	36.3	33.8	50.7	54.3

BAR, Barisal; CTG, Chittagong; DHK, Dhaka; KHL, Khulna; RAJ, Rajshahi; RNG, Rangpur; SYL, Sylhet; BD, Bangladesh.

For water and sanitation indicators, cluster 2 (19 districts) were well below the national level in terms of safe disposal of child feces and availability of water and soap at the place of hand washing (S2 Table, S2 Fig).

**Fig 5:**
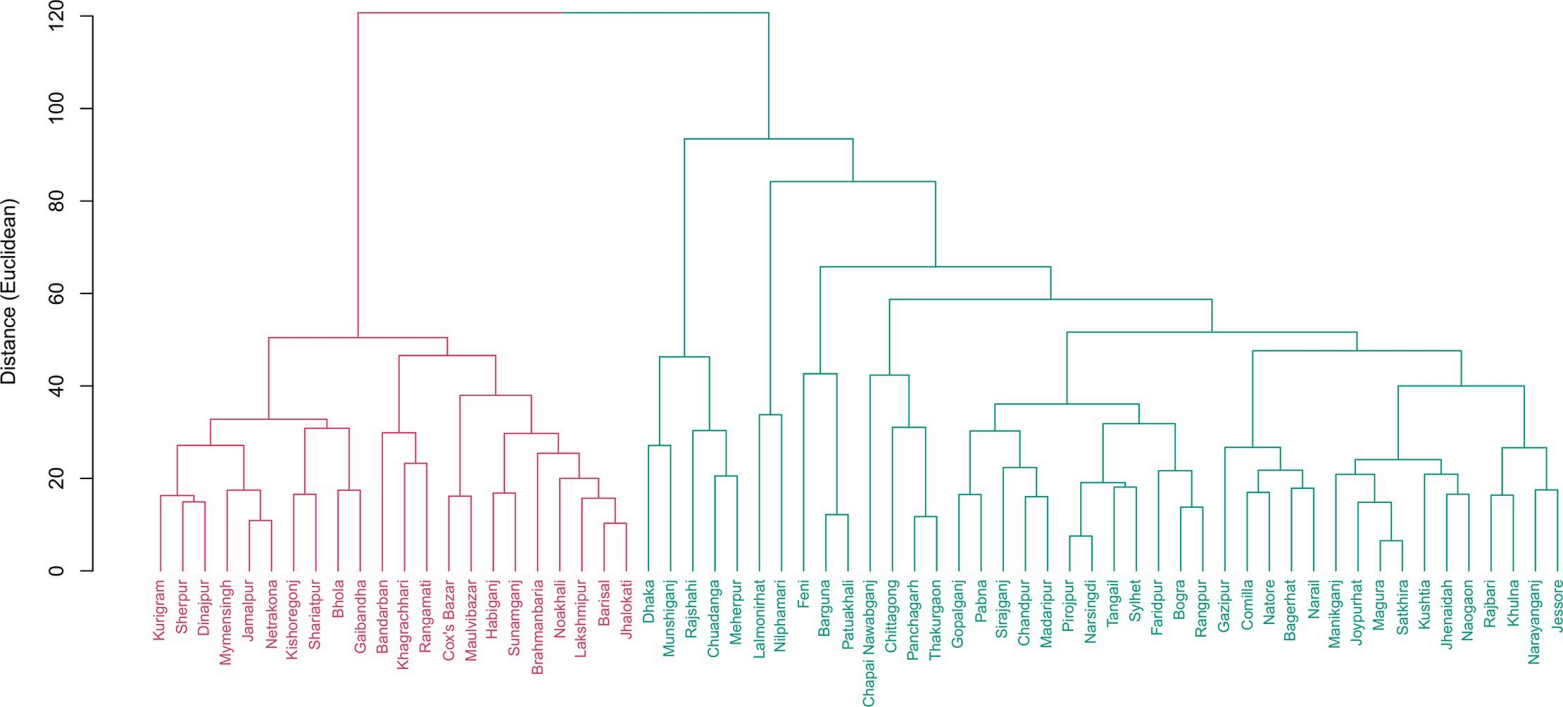
Cluster of districts based on reproductive health indicators. Two clusters were found. Cluster 1 include 42 districts while 22 districts were in cluster 2. Districts in cluster 2 have much lower antenatal care coverage (at least once by skilled health professional) (43.4% vs 58.7% nationally), lower percentage of skilled attendant at delivery (26% vs 43.5% nationally), nearly half on institutional delivery (16.1% vs 31% nationally).

Table 4 and Fig 5 represent clusters and cluster averages for reproductive health indicators. Districts in cluster 2 (22 districts) had higher TFR per thousand women compared to the national rate (2.6 vs 2.3) whereas the other 42 districts had lower TFR (2.1). Most noticeably, districts in cluster 2 had much lower antenatal care coverage (at least once by skilled health professional) (43.4% vs 58.7% nationally), lower percentage of skilled attendant at delivery (26% vs 43.5% nationally), nearly half on institutional delivery (16.1% vs 31% nationally). However, these districts had much lower cesarean delivery compared to national figures (9.1% vs 19.1% nationally).

**Table 4:** Cluster averages of districts along with averages of the divisions and Bangladesh as a whole based on reproductive health indicators.

	Cluster Average									
Indicators	Cluster 1	Cluster 2								
	42 districts	22 districts	BAR	CTG	DHK	KHL	RAJ	RNG	SYL	BD
TFR per 1000 women	2.1	2.6	2.3	2.7	2.3	1.9	1.9	2.2	2.9	2.3
Early childbearing before age 18	24.9	26.1	21.3	19.7	23.3	27.1	34.0	30.5	14.8	24.4
Contraceptive prevalence rate—any method	63.6	61.3	56.8	53.0	60.1	70.3	68.1	72.9	46.5	61.8
Unmet need	12.4	15.2	19.0	18.0	15.0	9.3	10.2	9.1	16.3	13.9
Antenatal care: at least once by skilled health professional	65.1	43.4	40.3	58.1	61.9	74.6	63.6	46.6	52.1	58.7
Antenatal care coverage: at least four times by any provider	28.8	12.7	14.0	21.7	26.3	27.1	25.6	35.8	16.0	24.7
Skilled attendant at delivery	52.3	26.0	38.4	41.5	44.8	56.7	51.8	39.4	26.7	43.5
Institutional delivery	37.5	16.1	17.1	27.1	34.9	45.6	38.1	23.0	20.8	31.0
Cesarean delivery	23.7	9.1	10.5	14.5	24.4	30.5	22.4	11.7	10.8	19.1

BAR, Barisal; CTG, Chittagong; DHK, Dhaka; KHL, Khulna; RAJ, Rajshahi; RNG, Rangpur; SYL, Sylhet; BD, Bangladesh.

Mass media and HIV/AIDS awareness indicators are represented in S3 and S4 Tables and S3 and S4 Figs. Cluster-2 with 5 districts were well above the national averages on all the media awareness indicators where these five districts have nearly twice as much (12.4% vs 6.1% nationally) women aged 15–24 who used computer during last 12 months (S3 Table). Three clusters were found to best represent the 64 districts: 36 in cluster 1, 10 in cluster 2 and 18 in cluster 3 (S4 Fig) for HIV/AIDS awareness indicators. Cluster-1 showed averages that were well below the other two clusters as well as national figures. In particular, percentages of women who heard of AIDS (39.2%), had knowledge about HIV prevention (6.9%), and knowledge about mother-to-child transmission (17%) were much lower in these 36 districts compared to the rest of the country (55.8%, 9.1%, 21.7%, respectively) (S4 Table).

## Discussion

We found that Bangladesh is more or less homogeneous based on percent male-headed households, unemployment rate, average household size, and population density per square kilometer. Bangladesh has achieved some success in terms of birth registration with approximately 37% of births registered of children under age 5. But the percentage is much lower than South Asian region (about 62%) and almost half of the global figure (about 71%) [15]. We found six districts namely, Narsingdi, Chapai Nawabganj, Laksmipur, Rangpur, Sherpur, and Nilphamari to be lagging behind rest of the country on this indicator by more than 10 percentage points (Fig 2). Notably, four of these districts are located in the North and North-west part of Bangladesh. Interestingly, these districts have more than 10 points higher percentage of women (15–49) married before age 15 compared to rest of the country (34% vs 24% nationally).

Early initiation of breastfeeding within one hour of birth refers to the best practice recommendation by the world health organization (WHO) [16]. In South Asia, merely 39% of newborns are breastfed within one hour of birth whereas the global prevalence is 44%. However, the proportion of early initiation of breastfeeding is better in Bangladesh (57.4%) compared to the South Asia as well as global prevalence [15]. There is little difference in the pattern of breastfeeding indicators across the country. Overall, 11 districts namely, Jhalokati, Feni, Jamalpur, Narail, Cox’s Bazar, Chittagong, Meherpur, Pirojpur, Kurigram, Natore, and Lalmonirhat are way behind national average in terms of number of babies breastfed within one hour of birth. Of them, Pirojpur have the lowest percentage with only 13.1% babies who were breastfed within one hour of birth. The reproductive health indicators are important indicators for assessing the condition of maternal health in Bangladesh. 22 districts are well below the national level when it comes to antenatal care coverage, skilled attendant at delivery and institutional delivery practices. These districts need to be brought up to par with the rest of the country. There are 19.1% of births in Bangladesh are through cesarean delivery which is above the “medically necessary” target of 10%-15% that WHO suggests is ideal [17]. However, the average is much higher in majority of the districts (41 out of 64) where the rate is 23% compared to national figure of 19.1%. Interestingly, the low performing districts have only 9.1% of births through cesarean delivery which is near about WHO suggested threshold. The contrast is perhaps due to potential association between better access to antenatal care and the choice of delivery mode. Globally coverage of skilled attendant during childbirth increased from 62% in 2000 to 73% in 2013 [18], but Bangladesh is far behind with a prevalence of 43.5% nationally. In addition, the percentage of births that take place in a health facility in Bangladesh is much lower than the global estimate of about two-thirds [19].

However, Bangladesh has made considerable progress in institutional delivery rates from 4 to 29% between 1993 and 2011 [20]. On the literacy related indicators, five districts are well above the national averages. These are Dhaka, Khulna, Faridpur, Munsiganj, and Rajshahi. Of them, Dhaka, Khulna and Rajshahi are divisional headquarters. Notably absent are the other divisional headquarters–Chittagong, Barisal, and Sylhet in the cluster.

**Fig 6:**
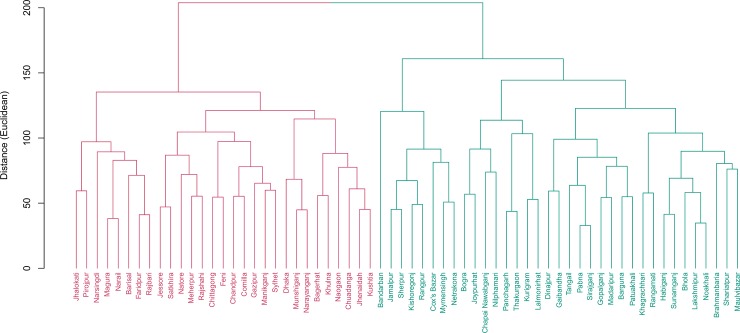
Cluster of districts based on all indicators combined. There are 29 districts in cluster 1 and 35 districts in cluster 2.

**Fig 7:**
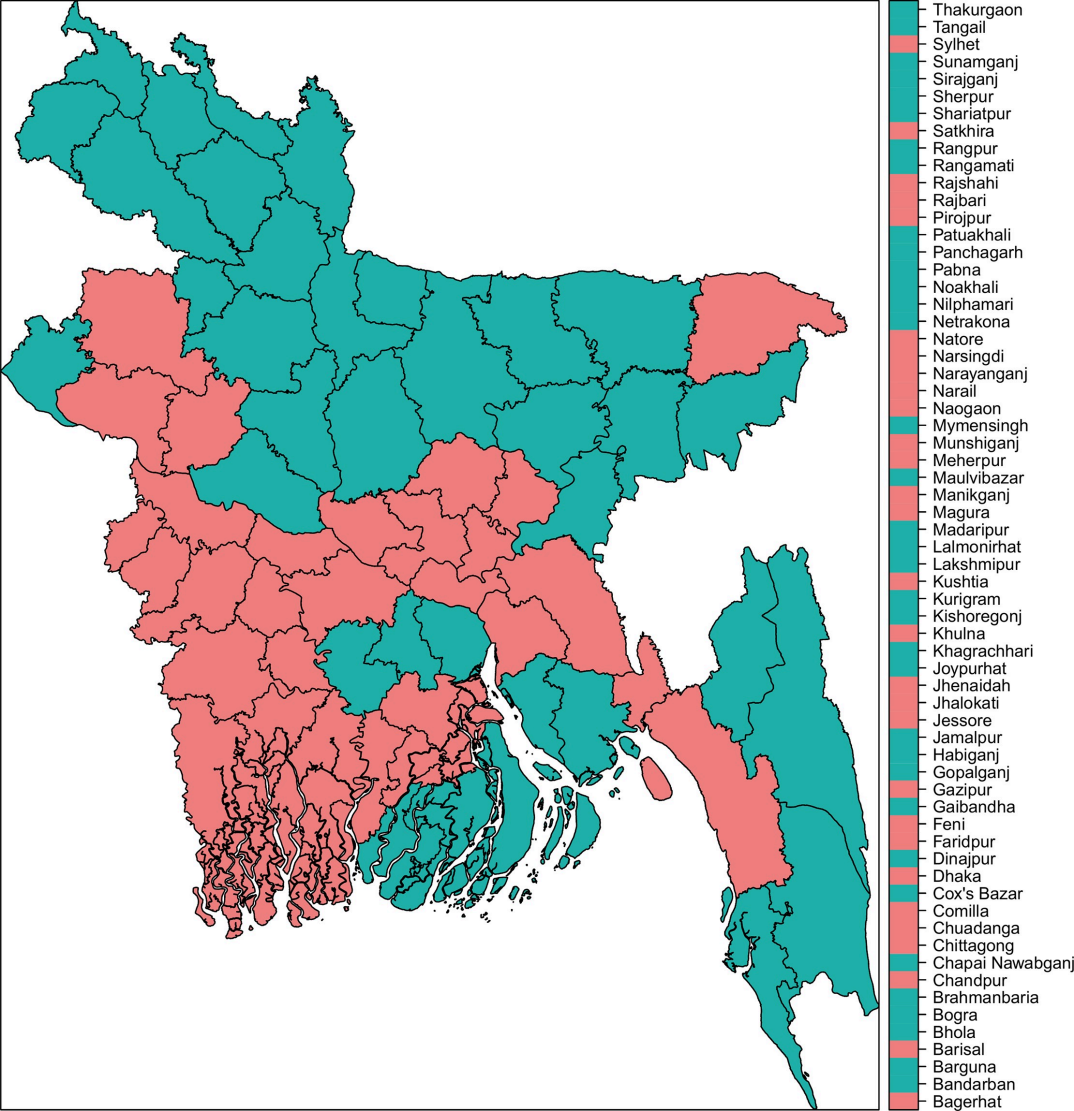
Regional distribution of the clustered districts based on all indicators combined. The map depicted in this figure is our own. Under-performing districts (red) are mostly from the North and Northeastern part while the majority of the well-performing districts are in the central and Southwestern parts of Bangladesh.

Comprehensive knowledge of HIV/AIDS is not prevalent in Bangladesh. Among the female adolescents (15–19 years of age), this number is about 10%, which is almost half of the global prevalence. Moreover, the urban- rural and richest-poorest ratio of comprehensive knowledge of HIV/AIDS among females aged 15–24 years are 1.8% and 8.9% in Bangladesh [15]. Based on all the indicators together, Bangladesh can be divided into two distinct clusters with 35 and 29 districts in each. See Figs 6 and 7 for the districts in each cluster. Underperforming districts are mostly from the North and Northeastern regions while the majority of the well- performing districts are in the central and Southwestern parts of Bangladesh. Chittagong hill tracts and some southern districts also fell under the poorly performing regions. Our analysis reveals that the differences in the coverage of maternal and child health indicators are striking at the sub-national level in Bangladesh. Between-districts variability is clearly visible for majority of the indicators, with a few exceptions. The observed difference between the high-performing region and low-performing region demands region- specific interventions and program recommendations. All indicators have some geo-spatial association which is not surprising because of the socio-cultural influences that may exist for many of the maternal and child health indicators studied, especially breastfeeding, malnutrition, contraceptive use, antenatal care etc.

To respond to the regional disparities, refocusing on the health systems on the accessibility and affordability to quality services for populations of disadvantaged areas and to implement monitoring mechanism to check the progress over time is needed. In addition, geospatial tools could be implemented as part of interventions for monitoring the activities, coverage and variations to identify the hotspots and deploy resources accordingly. Moreover, it is necessary to design program and interventions and their performances by different stratifications (such as rural, urban and urban slum; rich and poor, geographically hard to reach areas and non-hard to reach areas etc.). Awareness among health service providers on critical issues specifically effective newborn care, reproductive health and quality health services, knowledge and attitudinal barriers to good health practices as well as their benefits among household members are also needed to improve through effective interventions and social advocacy which may help to reduce regional gaps. Disparities also demand focusing on the decentralization of national level health planning and budgeting to optimize the sub-national level health outcomes in Bangladesh.

This study has some limitations, which include the inability to compare individual district or division on the chosen indicators. However, our findings would supplement the existing summary in a more comprehensive way. Future research could be directed towards developing a composite index based on the underlying indicators.

## Conclusions

In this study, we used maternal and child health indicators to study regional disparities in Bangladesh using a multivariate approach. Our findings have divided overall Bangladesh into two clusters based on different health related indicators, except for the HIV/AIDS awareness indicators which produced three clusters. Interestingly, districts in different clusters were not the same for all set of indicators which indicates the presence of regional variation across the indicators. Socioeconomic and cultural characteristics vary notably across the districts, but clustering pattern shows how similar different districts are within the clusters.

This study findings provide materials for policy makers to obtain a summarized assessment of the characteristics of different districts in Bangladesh with respect to important maternal and child health indicators. This would allow them to formulate necessary policies and to implement development strategies. In summary, the findings suggest the need to differentially focus on community-level interventions aimed at increasing maternal and child health care utilization and improving the socioeconomic position of mothers, especially in the disadvantaged districts in Bangladesh.

## Supporting information

S1 Fig Cluster of districts based on breastfeeding indicators. Two clusters were found. Cluster 1 include 53 districts while 11 districts were in cluster 2. Percentage of babies breastfed within one hour is nearly 50% less in these 11 districts than the corresponding national figure.
(TIF)

S2 Fig Cluster of districts based on water and sanitation indicators. Two clusters were found. Cluster 1 include 45 districts while 19 districts were in cluster 2. Districts in cluster 2 are performing below national averages.
(TIF)

S3 Fig Cluster of districts based on media awareness indicators. Two clusters were found. Cluster 1 include 59 districts while 5 districts were in cluster 2. Districts in cluster 2 are well above national averages.
(TIF)

S4 Fig Cluster of districts based on HIV/AIDS awareness indicators. Three clusters were found. Cluster 1 include 36 districts, where 10 and 18 districts were in cluster 2 and cluster 3, respectively. Districts in cluster 1 show much lower percentages on the indicators compared to the other two clusters as well as national figures.
(TIF)

S1 Table Cluster averages of districts along with averages of the divisions and Bangladesh as a whole based on breastfeeding indicators. (DOCX)

S2 Table Cluster averages of districts along with averages of the divisions and Bangladesh as a whole based on water and sanitation indicators. (DOCX)

S3 Table Cluster averages of districts along with averages of the divisions and Bangladesh as a whole based on mass media awareness indicators. (DOCX)

S4 Table Cluster averages of districts along with averages of the divisions and Bangladesh as a whole based on HIV/AIDS awareness among women 15–49. (DOCX)
