# Supplementary figures and images for: Regional disparities in maternal and child health indicators: Cluster analysis of districts in Bangladesh
Source: PLoS One. 2019 Feb 6;14(2):e0210697. doi: 10.1371/journal.pone.0210697 (PMC6364878; doi:10.1371/journal.pone.0210697)

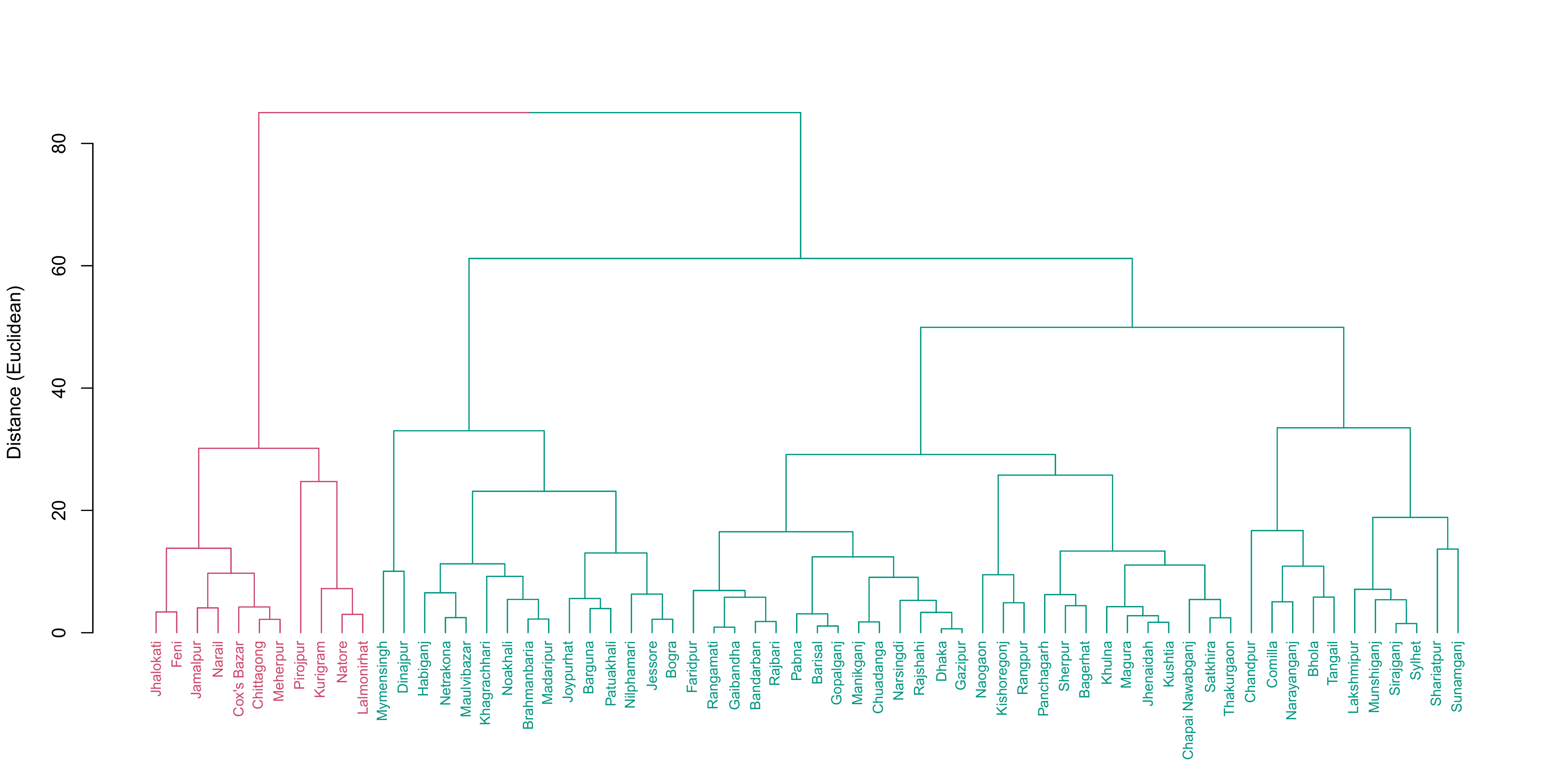

Supplement: S1 Fig — Two clusters were found. Cluster 1 include 53 districts while 11 districts were in cluster 2. Percentage of babies breastfed within one hour is nearly 50% less in these 11 districts than the corresponding national figure. (TIF) [file pone.0210697.s001.tif]

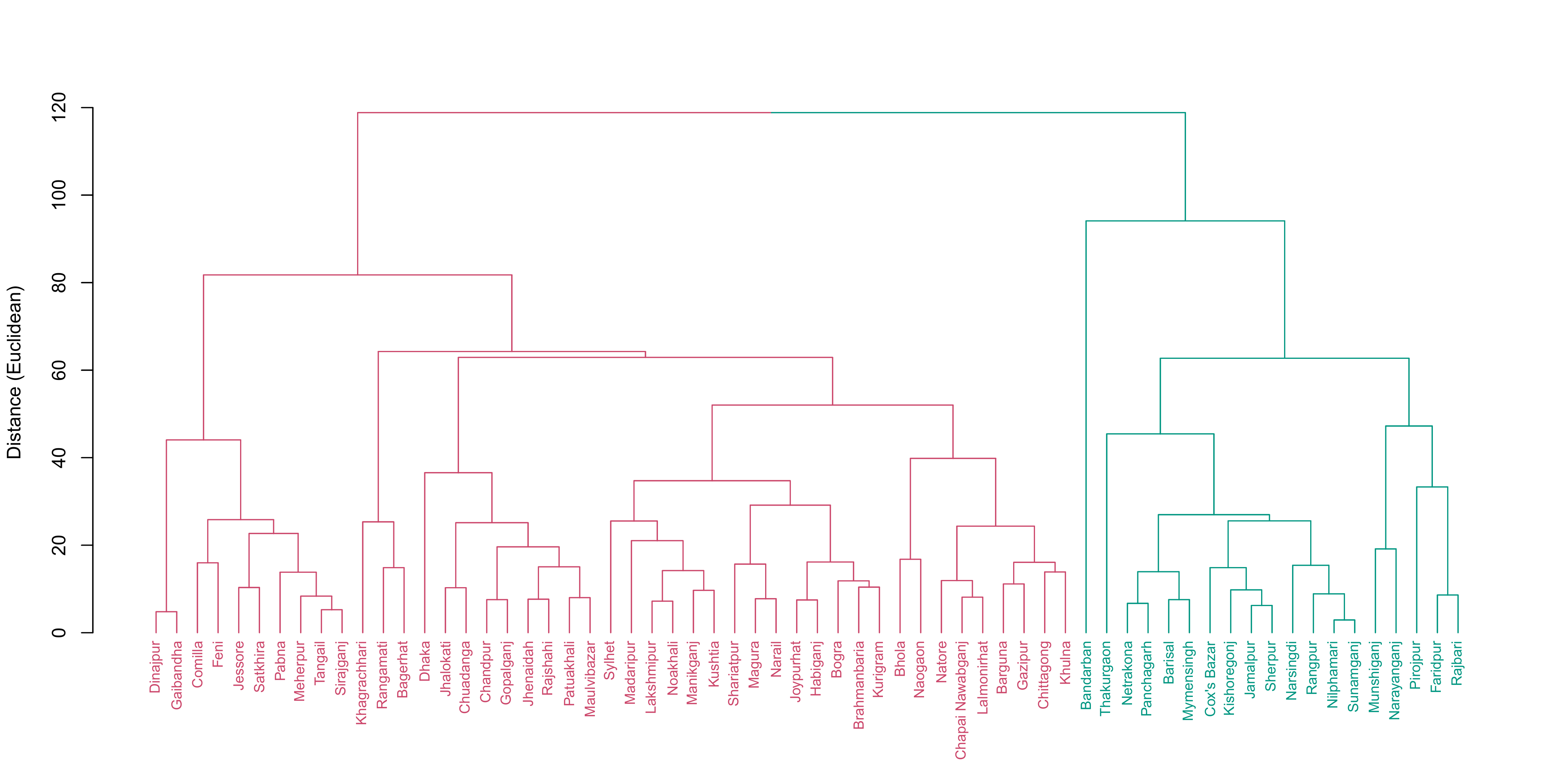

Supplement: S2 Fig — Two clusters were found. Cluster 1 include 45 districts while 19 districts were in cluster 2. Districts in cluster 2 are performing below national averages. (TIF) [file pone.0210697.s002.tif]

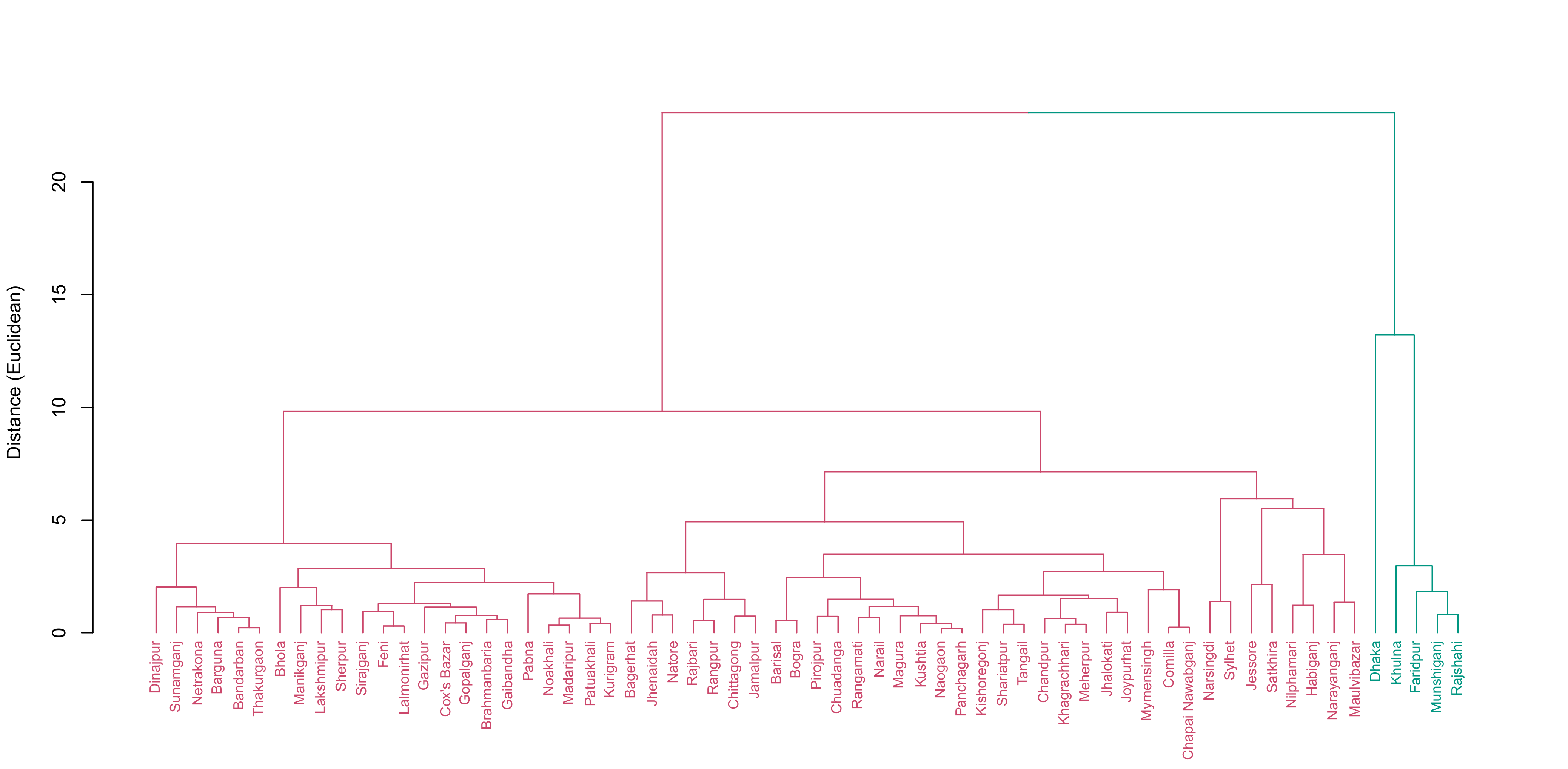

Supplement: S3 Fig — Two clusters were found. Cluster 1 include 59 districts while 5 districts were in cluster 2. Districts in cluster 2 are well above national averages. (TIF) [file pone.0210697.s003.tif]

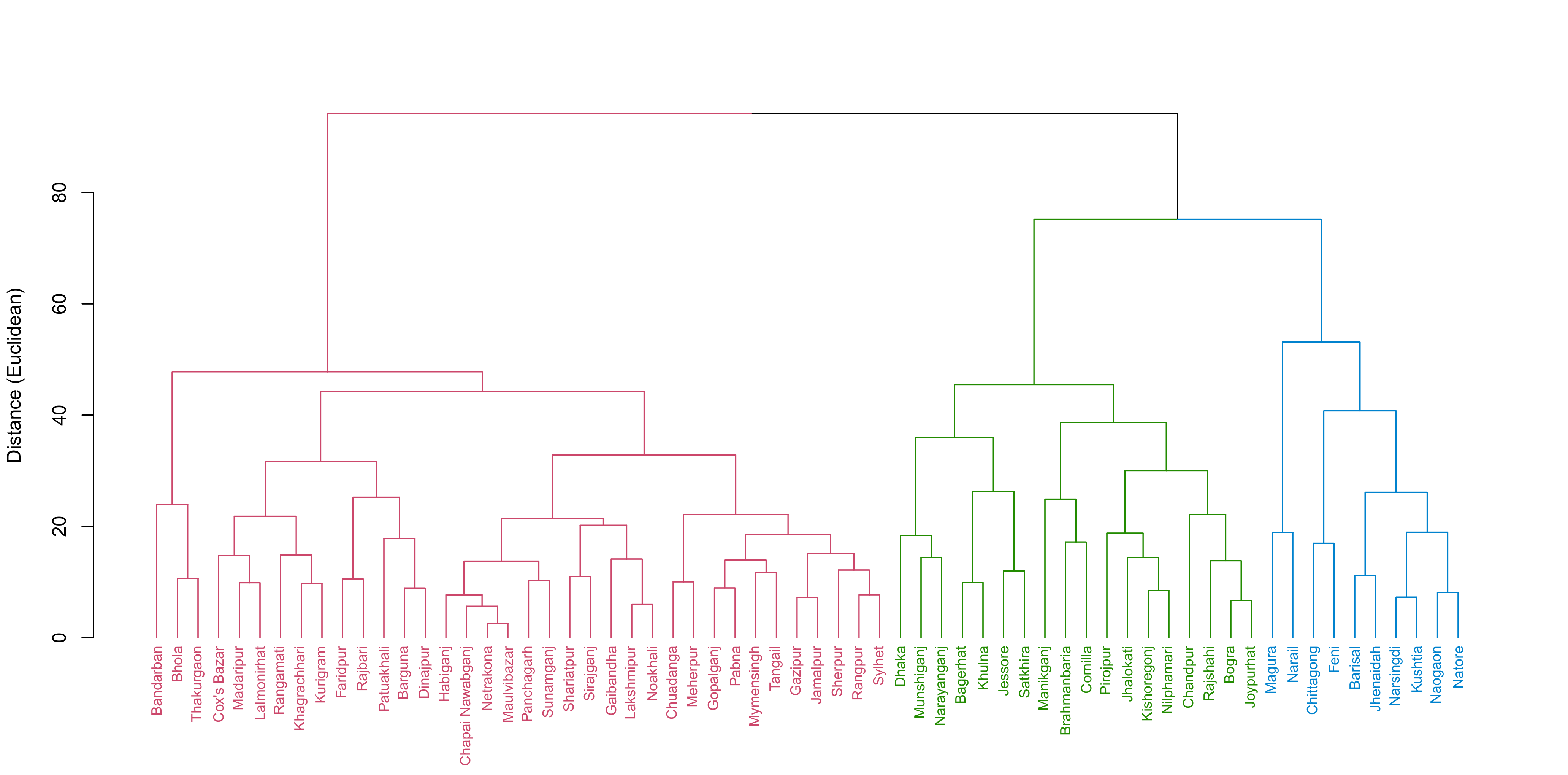

Supplement: S4 Fig — Three clusters were found. Cluster 1 include 36 districts, where 10 and 18 districts were in cluster 2 and cluster 3, respectively. Districts in cluster 1 show much lower percentages on the indicators compared to the other two clusters as well as national figures. (TIF) [file pone.0210697.s004.tif]
