# Supplementary material for: Regional disparities in maternal and child health indicators: Cluster analysis of districts in Bangladesh
Source: PLoS One. 2019 Feb 6;14(2):e0210697. doi: 10.1371/journal.pone.0210697 (PMC6364878; doi:10.1371/journal.pone.0210697)
Supplement: S1 Table — (DOCX) [file pone.0210697.s005.docx]

**S1 Table. Cluster averages of districts along with averages of the divisions and Bangladesh as a whole based on breastfeeding indicators.**

| **Indicators** | **Cluster Average** | |  |  |  |  |  |  |  |  |
| --- | --- | --- | --- | --- | --- | --- | --- | --- | --- | --- |
|  | **Cluster 1** | **Cluster 2** |  |  |  |  |  |  |  |  |
|  | **53 districts** | **11 districts** | **BAR** | **CTG** | **DHK** | **KHL** | **RAJ** | **RNG** | **SYL** | **BD** |
| Breastfed within one hour of birth | 60.8 | 33.9 | 55.8 | 53.1 | 59.6 | 47.3 | 56.5 | 59.1 | 73.5 | 57.4 |
| Children 0-23 months with age appropriate breastfeeding | 68.3 | 66.9 | 62.0 | 58.3 | 65.5 | 76.2 | 72.9 | 76.8 | 63.0 | 66.5 |

BAR, Barisal; CTG, Chittagong; DHK, Dhaka; KHL, Khulna; RAJ, Rajshahi; RNG, Rangpur; SYL, Sylhet; BD, Bangladesh.
