# Supplementary material for: Regional disparities in maternal and child health indicators: Cluster analysis of districts in Bangladesh
Source: PLoS One. 2019 Feb 6;14(2):e0210697. doi: 10.1371/journal.pone.0210697 (PMC6364878; doi:10.1371/journal.pone.0210697)
Supplement: S2 Table — (DOCX) [file pone.0210697.s006.docx]

**S2 Table. Cluster averages of districts along with averages of the divisions and Bangladesh as a whole based on water and sanitation indicators.**

|  | **Cluster Average** | |  | | | | | | | |
| --- | --- | --- | --- | --- | --- | --- | --- | --- | --- | --- |
| **Indicators** | **Cluster 1** | **Cluster 2** |  |  |  |  |  |  |  |  |
|  | **45 districts** | **19 districts** | **BAR** | **CTG** | **DHK** | **KHL** | **RAJ** | **RNG** | **SYL** | **BD** |
| Using improved drinking water sources | 96.2 | 95.8 | 95.3 | 97.0 | 99.9 | 94.4 | 99.3 | 99.9 | 93.8 | 97.9 |
| Improved sanitation which are not shared | 58.4 | 47.2 | 52.0 | 59.4 | 54.0 | 58.0 | 52.0 | 57.4 | 58.6 | 55.9 |
| Safe disposal of child feces | 38.9 | 31.2 | 39.3 | 37.6 | 46.0 | 43.8 | 34.6 | 21.4 | 35.5 | 38.7 |
| Water and soap available at place of hand washing | 68.0 | 26.7 | 50.0 | 71.4 | 50.1 | 66.5 | 67.9 | 61.5 | 54.2 | 59.1 |

BAR, Barisal; CTG, Chittagong; DHK, Dhaka; KHL, Khulna; RAJ, Rajshahi; RNG, Rangpur; SYL, Sylhet; BD, Bangladesh.
