# Supplementary material for: Regional disparities in maternal and child health indicators: Cluster analysis of districts in Bangladesh
Source: PLoS One. 2019 Feb 6;14(2):e0210697. doi: 10.1371/journal.pone.0210697 (PMC6364878; doi:10.1371/journal.pone.0210697)
Supplement: S3 Table — (DOCX) [file pone.0210697.s007.docx]

**S3 Table. Cluster averages of districts along with averages of the divisions and Bangladesh as a whole based on mass media awareness indicators.**

|  | **Cluster Average** | |  | | | | | | | |
| --- | --- | --- | --- | --- | --- | --- | --- | --- | --- | --- |
| **Indicators** | **Cluster 1** | **Cluster 2** |  |  |  |  |  |  |  |  |
|  | **59 districts** | **5 districts** | **BAR** | **CTG** | **DHK** | **KHL** | **RAJ** | **RNG** | **SYL** | **BD** |
| Read/watch newspaper, magazine, ratio, tv | 0.9 | 2.5 | 0.8 | 0.6 | 2.3 | 2.1 | 1.0 | 0.9 | 2.7 | 1.6 |
| Women (15-24) used computer during last 12 months | 4.2 | 12.4 | 3.4 | 4.9 | 8.8 | 6.8 | 5.4 | 3.3 | 4.5 | 6.1 |
| Women (15-24) used Internet during last 12 months | 1.6 | 6.4 | 0.9 | 2.1 | 5.3 | 2.0 | 1.7 | 1.4 | 3.3 | 3.0 |

BAR, Barisal; CTG, Chittagong; DHK, Dhaka; KHL, Khulna; RAJ, Rajshahi; RNG, Rangpur; SYL, Sylhet; BD, Bangladesh.
