# Supplementary material for: Regional disparities in maternal and child health indicators: Cluster analysis of districts in Bangladesh
Source: PLoS One. 2019 Feb 6;14(2):e0210697. doi: 10.1371/journal.pone.0210697 (PMC6364878; doi:10.1371/journal.pone.0210697)
Supplement: S4 Table — (DOCX) [file pone.0210697.s008.docx]

**S4 Table. Cluster averages of districts along with averages of the divisions and Bangladesh as a whole based on HIV/AIDS awareness among women 15-49.**

|  | **Cluster Average** | | |  |  |  |  |  |  |  |  |
| --- | --- | --- | --- | --- | --- | --- | --- | --- | --- | --- | --- |
| **Indicators** | **Cluster 1** | **Cluster 2** | **Cluster 3** |  |  |  |  |  |  |  |  |
|  | **36 districts** | **10 districts** | **18 districts** | **BAR** | **CTG** | **DHK** | **KHL** | **RAJ** | **RNG** | **SYL** | **BD** |
| Women heard of AIDS | 39.2 | 69.9 | 69.8 | 50.2 | 58.1 | 58.6 | 76.6 | 52.4 | 38.3 | 42.8 | 55.8 |
| Knowledge about HIV prevention | 6.9 | 12.6 | 9.8 | 7.4 | 5.5 | 13.0 | 9.4 | 10.0 | 5.9 | 5.3 | 9.1 |
| Knowledge about mother-to-child transmission | 17.0 | 43.9 | 20.3 | 26.1 | 20.4 | 19.8 | 32.9 | 25.2 | 15.3 | 14.0 | 21.7 |
| Accepting attitude towards people living with AIDS | 37.9 | 47.5 | 27.2 | 39.9 | 41.4 | 40.2 | 29.8 | 37.1 | 33.8 | 28.1 | 37.2 |
| Women know where to get tested for HIV | 7.2 | 10.6 | 15.4 | 8.2 | 13.8 | 11.0 | 16.4 | 10.5 | 8.5 | 6.2 | 11.3 |
| Women with HIV counseling during antenatal care | 1.7 | 2.3 | 2.4 | 1.4 | 2.8 | 2.6 | 2.7 | 2.2 | 1.9 | 2.8 | 2.5 |

BAR, Barisal; CTG, Chittagong; DHK, Dhaka; KHL, Khulna; RAJ, Rajshahi; RNG, Rangpur; SYL, Sylhet; BD, Bangladesh.
